# As It Stands: The Palouse Wild Cider Apple Breeding Program

**DOI:** 10.3390/plants11040517

**Published:** 2022-02-14

**Authors:** Tymon James, Alexandra Johnson, Alexander Schaller, Stijn Vanderzande, Feixiong Luo, Paul Sandefur, Sushan Ru, Cameron Peace

**Affiliations:** 1 Department of Horticulture, Washington State University, Pullman, WA 99164, USA; tymon.james@wsu.edu (T.J.); alexandra.johnson2@wsu.edu (A.J.); stijn.vanderzande@wsu.edu (S.V.); 2 Department of Environmental Horticulture, University of Florida, Gainesville, FL 32611, USA; aschaller@ufl.edu; 3 Department of Pomology, Hunan Agricultural University, Changsha 410128, China; flybearluofeixiong@126.com; 4 Fall Creek Farm and Nursery, Inc., Lowell, OR 97452, USA; pauls@fallcreeknursery.com; 5 Department of Horticulture, Auburn University, Auburn, AL 36849, USA; szr0099@auburn.edu

**Keywords:** crop wild relatives, DNA information, experiential learning, germplasm

## Abstract

Providing hands-on education for the next generation of plant breeders would help maximize effectiveness of future breeding efforts. Such education should include training in introgression of crop wild relative alleles, which can increase genetic diversity while providing cultivar attributes that meet industry and consumer demands in a crop such as cider apple. Incorporation of DNA information in breeding decisions has become more common and is another skill future plant breeders need. The Palouse Wild Cider apple breeding program (PWCabp) was established at Washington State University in early 2014 as a student-run experiential learning opportunity. The objectives of this study were to describe the PWCabp’s approaches, outcomes, and student involvement to date that has relied on a systematic operational structure, utilization of wild relatives, and incorporation of DNA information. Students chose the crop (cider apple) and initial target market and stakeholders (backyard growers and hobbyists of the Palouse region). Twelve target attributes were defined including high phenolics and red flesh. Phase one and two field trials were established. Two promising high-bitterness selections were identified and propagated. By running the PWCabp, more than 20 undergraduate and graduate students gained experience in the decisions and operations of a fruit breeding program. PWCabp activities have produced desirable new germplasm via utilization of highly diverse *Malus* germplasm and trained new plant breeding professionals via experiential learning.

## 1. Introduction

Food insecurity is a global issue that can be addressed efficiently, economically, and sustainably through plant breeding encompassing training the next generation of plant breeders in the use of modern breeding tools and strategies. As a process, plant breeding consists of genetically improving crops over time via controlling inputs (parents) to achieve desired outputs (superior seedlings) that meet the needs of changing commercial and consumer desires as well as pressing global issues such as climate change, disease pressure, and population increase [1,2,3]. Addressing ever-evolving challenges means that breeding new, superior cultivars is a continuous necessity. The multidisciplinary nature of plant breeding coupled with the likelihood that breeders will conduct diverse professional activities has obviated the need for breadth and depth in a plant breeder’s education [4,5]. However, if envisioned improvements in crop productivity, stress tolerance, resource utilization, and product quality are to be realized, plant breeders that are educated in modern scientific techniques and knowledgeable about local production practices must be prepared to meet ongoing challenges and opportunities [6]. Developing professionals that are prepared out of college to benefit any breeding program and that can help confront current challenges of the plant breeding field is a need not yet fulfilled.

Plant breeding is an applied science and is therefore best taught interactively. Prospective breeders need tangible, hands-on experiences to learn from for future breeding efforts to be as effective and successful as possible [7]. Experiential learning connects theoretical concepts learned in the classroom to empirical results observed in the field in a meaningful, real-world way. Active, hands-on involvement has been reported to improve student learning outcomes, specifically acquisition and utilization of new knowledge, skills, and attitudes [8].

Useful to aspiring plant breeder’s future careers is education and hands-on experience in the use of crop wild relatives (CWR) for maintaining and increasing genetic diversity within a crop while incorporating desirable, novel CWR alleles into commercial cultivars. Domestication has reduced or eliminated genetic diversity at some genomic regions in modern crops, constricting the potential for developing cultivars with improved traits [9]. In contrast, crop wild relatives retain high levels of genetic diversity compared to their domesticated descendants [10]. Introgression or incorporation of alleles from wild sources into a new individual via breeding can increase useful genetic diversity and provide sets of cultivar attributes that help meet industry and consumer demands [11,12]. The achievements of breeding programs that have successfully exploited wild perennial species are not yet widespread [13], and therefore knowledge and experience with introgression strategies will be valuable to any prospective plant breeder’s career.

DNA-informed breeding holds promise for targeted crop improvement and is currently a critical approach for future breeders to have in their repertoire. The skills of obtaining and interpreting genotypic data are crucial for enabling DNA-informed breeding. Through genomic characterization, breeding materials’ identity is confirmed, and relatedness is estimated [14,15,16]. Marker-assisted selection (MAS) is performed by running diagnostic, trait-specific DNA tests on prospective parents and seedlings in a breeding program. Individual plants harboring the alleles deemed desirable in the program are selected as parents or advanced as selections in the case of seedlings. Conversely, seedlings found to harbor undesirable alleles can be culled. MAS identifies the genetic potential for targeted traits in parents and seedlings, enabling more efficient use of breeding program resources [17]; it is becoming more common and even conventional in rosaceous crop breeding [18,19,20,21]. Extensive genetic information useful for DNA-informed breeding is available on many crops. For example, in apple (*M*. *domestica*), traits influenced heavily by genetics such as disease resistance, growth habit, fruit quality traits such as phenolics content, and many others have been studied on large sets of apple individuals with genetic data and DNA tests developed for such traits using markers such as SSRs and SNPs [20,21]. Most students lack the opportunity to gain experience with approaches such as DNA-informed breeding in the safety of a learning environment when career-affecting consequences are not at stake.

Future plant breeders need to gain experience in overall understanding of the structure of breeding operations. Planning and organizing the many and varied activities that need to occur in a breeding program makes targeted cultivar development more manageable. A useful way for breeders to plan, manage, and organize activities is to put them into the four workflow stages defined [19] as (1) goal-setting: ensuring program targets align with industry, stakeholder, market, and consumer needs and desires; determining what sets of attributes to target; (2) obtaining and creating new genetic variation: usually through controlled crossing, combining new sets of parental alleles into single individuals; (3) selection: an approach such as DNA-informed breeding is used to determine and choose individuals that have the best genetic potential for advancement; (4) commercialization: proving cultivars are distinct and new; clonal propagation to large numbers suitable for commercial production and convincing growers to buy and plant them. Conceptualizing breeding program organization into such components would help in preparing plant breeding students to plan and conduct activities contributing to cultivar development free from the idiosyncrasies of any particular program in which they might train.

Training the plant breeders of tomorrow in new cultivar development via access to diverse genetic resources and application of DNA information, within a generic operational framework, is appropriate for a higher learning institution of the U.S. university land grant system [22] such as Washington State University (WSU). WSU’s Department of Horticulture planted in 2010 highly diverse apple (*Malus* spp.) germplasm at its Pullman campus-adjacent Tukey Research Orchard (TRO) for the purpose of breeding education. The several hundred trees first established (“Diversity Set”) contained 1–4 trees of most accessions of the USDA National Plant Germplasm System’s apple core collection [23], including at least 25 CWR species, heritage and modern cultivars, and breeding selections. In the same year, more than 800 donated seedling trees (in their third year) of the apple crop progenitor species *M. sieversii* were also planted at TRO (“Sieversii Site 6 and 9 Set”). These trees had been growing in pots and were one of the replicate populations of pooled seeds generated by crossing among carefully chosen sets of *M. sieversii* accessions originally from two wild populations in Kazakhstan [24]. All of these genetic resources were maintained at TRO in anticipation of a new graduate course in which the inaugural class would establish the scope of the target industry and begin operations. A student-run breeding program was officially established at WSU in Pullman, WA, in early 2014 for the WSU course HORT 495/503 “Fruit Breeding for the New Millennium” to provide hands-on experience for plant breeding students. This “Palouse Wild Cider apple breeding program” (PWCabp) targeted cider apple for the Palouse region, a southeastern portion of Washington state that once had a viable apple industry [25] but is now well known for its highly productive non-irrigated wheat farming [26].

Cider apple is indeed a crop in need of genetic improvement. The U.S. cider apple industry is rapidly expanding as consumer demand increases but is based on outdated or unsuited cultivars [27]. Over the last decade, hard cider has been one of the fastest growing sectors in the craft beverage market, with a 2018 value of more than USD 2.2 billion including perry and rice wine segment [28]. Although cider-specific cultivars high in bitter-tasting compounds have existed for centuries, those used for modern cider production have many issues such as biennial bearing and a need for disease resistance due to a lack of targeted breeding. Much of the world’s cider industry relies on inferior fruit harvested from dessert cultivars but not meeting supermarket standards, and only rarely are cider-specific cultivars used [27,29]. Cider apple cultivars need improvements in biotic and abiotic stress resistance and other important production qualities such as non-biennial bearing to meet production demands [30]. Accessing apple’s genetic diversity by incorporating wild relatives in breeding might be useful in cider apple cultivar development. Apple wild relatives harbor novel alleles that can impart characteristics such as disease resistance and desirable cider quality attributes such as high bitterness and acidity [31,32]. The genetic improvements needed in cider apple have not yet been realized.

The PWCabp is intended to serve as a nexus for education in plant breeding involving utilization of wild germplasm and application of cutting-edge DNA-based diagnostics technologies. The objectives of this study were to describe the PWCabp’s approaches and outcomes to date in the context of the four operational stages while highlighting the use of wild apple relatives and DNA information in the program.

## 2. Results

### 2.1. Goal-Setting

At establishment of the PWCabp in 2014, the modest initial target of the program was decided: productive and hardy new cultivars for backyard growers and hobbyists of the local Palouse region, an area of rolling hills in eastern Washington, the distinctive fruit juice of which would provide a value-added boost when paired with juice of other cultivars. Four fruit attributes chosen to target were unusual type (e.g., red fleshed), high levels of phenolic compounds, providing a nutritional boost (e.g., high levels of anthocyanins), and low seediness, while eight tree attributes chosen included moderate to high resistance for the major pests and diseases of the region (e.g., fire blight), short juvenility, high yield, strong annual bearing tendency, spur-bearing habit, and attractive appearance (Table 1). In 2019, a systematic revision of target traits led to redefinition of four target fruit attributes and eight target tree attributes. Phenolic compound amount was refined to high bitterness (soft tannins) without high astringency (hard tannins). Juiciness was added to the fruit targets, while unusual type and nutritional boost were combined into flesh color (high levels of anthocyanins). Attractive tree appearance was split into leaf/bark appearance and branch angle. Fruit size thresholds were defined, and resistance to Cedar apple rust was added. Each defined trait level was given an “essential” or “enhancing” designation (Table 1).

### 2.2. New Genetic Variation

New genetic variation was created by utilizing widely diverse parents in new combinations and producing sufficient numbers of offspring to capture the desirable genetic variation harbored in parents. Students mostly chose parents with a wild background each year. Out of an average of 19 parents used each year, an average of nine wild or hybrid wild parents were used with 98% of families attempted involving at least one parent fully wild or with a recent wild background (Table 2). In 2013, most mothers chosen were among those trees growing and fruiting at the TRO. *M*. *sieversii* featured prominently and included the first 13 trees to be bearing fruit that year of the “Sieversii Site 6 and 9 Set” [24]. Other mothers were accessions of the crab apple cultivars (crab apple defined here as small-fruited, wild *Malus* species background) ‘Novosibirski Sweet’, ‘Kerr Crab’, ‘Robert’s Crab’, and the interspecific hybrid PRI E14-32. Fathers (solely from uncontrolled, open-pollination (OP) in the first year because breeding targets had not yet been defined) were expected to be nearby trees also growing in Block 17 of TRO (“Diversity Set”) and the *M*. *sieversii* seedling block (“Sieversii Site 6 and 9 Set”). Six *M*. *domestica* cultivars were used as mothers in early 2014 (to add to the 2013 cohort). Available fruit was collected from cold storage soon after the decision to focus on cider.

In the 2014 crossing season, controlled crossings were conducted for the first time, although some open-pollination-derived fruit was also collected to increase seed numbers (a tactic used in several years). The set of parents expanded genetic diversity to additional *Malus* species and prominently used a red-leaved/fleshed parent (‘Robert’s Crab’) (Table S1). Eleven specialty bittersweet/bittersharp cider cultivars were also used. In 2015, the parent pool was generally similar to that of 2014, but added several ornamental crab apple cultivars and used an increasing number of pairwise combinations. 2016-derived families were the most restricted, mostly consisting of the opportunistic acquisition of seedlings from a research project on powdery mildew resistance, with the crab-apple resistance sources ‘White Angel’, *M*. × *zumi*, *M*. × *robusta* “Persicifolia”, and *M*. *floribunda* crossed onto the dessert cultivars ‘Golden Delicious’ and ‘Fuji’. Other controlled crosses were from the North American *Malus* species *M*. *fusca* crossed with red-leaved/fleshed ‘Robert’s Crab’.

In 2017, second-generation parents were first added to the parent pool—these parents were fruiting seedlings of 2013 crosses such as the PWCabp- monikered “Big Blush” (*M*. *sieversii* background) and “Yellow Tiny” (*M*. *baccata* background)—and families created were mostly intercrosses among them. 2018 parents involved the first of the red-leaved/fleshed second-generation parents that arose from crosses in 2014 (PWCabp-monikered “Red 1” and “Red 2”), intercrossed with the most promising second-generation individuals first used as parents the year before. In 2019, the same second-generation parents were used again, now combined with larger-fruited *M*. *domestica* accessions, with pollen of many cider cultivars such as ‘Calville Rouge’, ‘Frequin’, and ’Amere de Berthcourt’ from Renaissance Orchards (Ferndale, WA). In 2020, several second-generation red-leaved/fleshed selections (PWCabp-monikered “Red 8” and “Red 11”) and high-bitterness selections from the program (PWCabp-monikered “Bitter Bomb”, “Bitter Cream”, and “Bitter Weeping”) were used as parents as well as several *M*. *domestica* cultivars. In 2021, PWCabp selections “Most Bitter”, “Bitter Cream”, “Bitter Weeping”, “Pink Puma”, “Red 7”, and “Red 2” were used as fathers crossed with several *M*. *domestica* cultivars including the cider apple cultivar ‘Kingston Black’. An average of 2000 seeds were created each season over eight years; however, over the most recent four years (2018–2021), this number averaged nearly 3000. Created seedlings have been flowering and fruiting since 2016, highlighting the successful capture of short juvenility phenotypes from parents (Table 2).

### 2.3. Selection

In successive years of PWCabp activities, students successfully germinated, greenhouse-raised, and field-planted seeds resulting from the previous year’s crosses. The greenhouse phase was effectively used in each year to reduce large numbers of created seedlings to a manageable set of vigorous, disease-free plants for the field. Students reduced a total of more than 17,000 seeds (average of 2000 per year) to approximately 2400 field-planted seedlings (average of 300 per year) (Table 2), a greenhouse-based selection intensity of 11% of plants kept. Genotyping of the first year’s open-pollination-derived families reduced almost 800 seedlings to 350 for field-planting; approximately one-third had an undesirable genotype for Ma-indel (i.e., homozygous for high- or low-acidity alleles), 10% for Rf-SSR (i.e., homozygous for no blush), and one-sixth for both DNA tests. Although genotypic information was obtained in 2019, no DNA-based-information was used due to missing the short window of opportunity for marker-assisted seedling selection while seedlings were in the greenhouse. Detailed greenhouse culling observations from 2019 and 2020 revealed that in both years, powdery mildew susceptibility was the main culling driver, reducing seedling numbers by 36% and 61% in 2019 and 2020, respectively. Culling for lack of resistance to apple cedar rust was higher in 2019 than 2020 at 36% and 15%, respectively (Tables S2 and S3).

Field trials were divided by students into two phases. Phase one was designated as the seedling field trial phase with a single tree per individual. Phase two was designated as the selection field trial phase with multiple trees per individual at multiple sites when possible. In 2019, students successfully redesigned and implemented the phase one trial for the new site at the WSU Horticulture Center. Chosen was an 85′ × 115′ rectangular plot on the southwest end of the new site. A four-year cycle was chosen for phase one, and thus the design split the seedlings into four groups. Each group consisted of four rows of 2 × 50 seedlings (400 total) (Figure S1). Students decided that in the first year, seedlings were to be planted in two-gallon fabric pots. In year two, seedlings were to be transplanted into 15-gallon fabric pots, where they spend their remaining time in this phase. Students designed the irrigation system that consisted of a manual valve on 1.5-inch flexpipe leading to two gallon per minute emitters in each pot (Figure S1).

Evaluation of the fruit of flowering seedlings in phase one at TRO from 2016 to 2021 resulted in identification of the high bitterness (bitterness score of 1.5 or higher) selections “Bitter Weeping” (bitterness: 1.0–1.5; astringency: 0; sweetness: 1.5; acidity: 0.5), “Bitter Cream” (bitterness: 2; astringency: 0.5; sweetness: 0.5; acidity: 0.5), and the red-fleshed selection “Pink Puma” (bitterness: 1.0; astringency: 0.5; sweetness: 1.0; acidity: 0.5). In 2019 and 2020, field-planted seedlings previously identified as suitable parents or selections because of their desirable phenotypes were successfully clonally propagated. In 2021, students successfully established the phase two cider trial row at the WSU Horticulture Center, resulting in the planting of two selections (“Bitter Weeping” and “Bitter Cream”) with 10 trees each, using five trees of ’Kingston Black’ (a standard cider cultivar) as a control for performance comparisons. The trial is expected to provide a sufficient amount of fruit that can be evaluated fresh or pressed, fermented, and evaluated for cider characteristics on a larger scale.

Target phenotypes such as short juvenility, red flesh, not biennial bearing, not fire blight-susceptible, and high bitterness were observed in some of the first seedlings from 2013 and 2014 crosses (Table S4). SNP array profiles for 18 PWCabp seedlings selected for advanced evaluation and/or used as parents revealed prominent incorporation of wild species such as *M*. *sieversii* and *M*. *baccata* (Table S4). Of the 12 OP-derived seedlings of 2013 and 2014 genome-scanned with the SNP array, 75% were revealed to have a non-*domestica* paternal parent. SNP array profile information also revealed that the paternal parent of “Big Blush” was the *M*. *domestica* cultivar ‘Haralson’, although the recorded mother of “Big Blush” (and its sibling “Bitter Bomb”) was determined to be incorrect. The open-pollination-derived “Red 5” and “Red 7” shared the paternal parent ‘Delicious’ (Table S4).

### 2.4. Commercialization

Members of the PWCabp have yet to identify selections possessing all required, defined trait levels to be advanced to the stage of commercialization.

### 2.5. Student Outcomes

**Figure 1:**
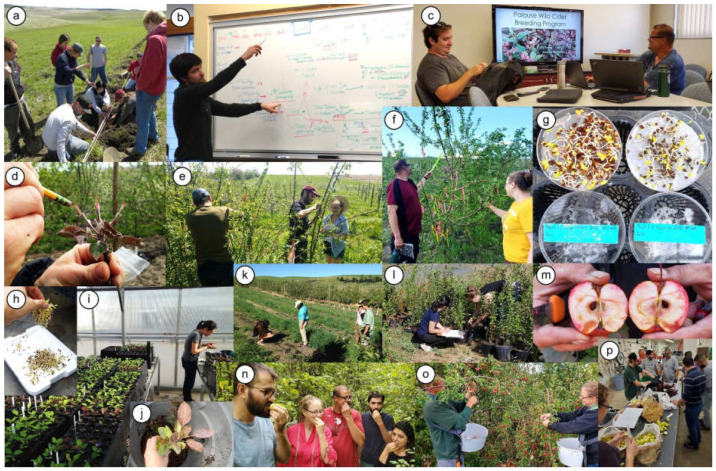
Experiential learning by students conducting operations in the Palouse Wild Cider apple breeding program. **Goal setting**: (**a**) planting in 2010 of the program’s base germplasm—grafted trees of most of the USDA National Plant Germplasm System’s apple core collection; (**b**) charting the breeding program’s course by the inaugural class of 2014, guided by trainee Paul Sandefur (P.S.); (**c**) reevaluating target attributes in early 2019, with trainees Alexander Schaller and Tymon James (T.J.) at the front of the classroom. **New genetic variation**: (**d**) pollinating emasculated flowers of ‘Robert’s Crab’ in spring 2015, from which it was realized that red-fleshed parents suit better as the father; (**e**) pollinating the selection “Bitter Shot” and an adjacent seedling in spring 2018 by trainees Saban Demir (S.D.), Ugur Emre (U.E.), and Alexandra Johnson (A.J.); (**f**) examining fruitlets forming on a heavily crossed tree of ‘Wealthy’ in summer 2021 by T.J. and A.J.; (**g**) germinating seeds in early 2019 of two 2018 families from crosses among some selected seedlings in the program that exhibited short juvenility. **Selection**: (**h**) discarding hundreds of germinated seeds in a family for which only those with red tissues were retained; (**i**) nurturing of greenhouse seedlings of 2014 families in early 2015 by trainee Hannah Walters (H.W.); (**j**) culling of a greenhouse seedling with a high incidence of powdery mildew infection; (**k**) examining newly planted seedlings of 2013 families in spring 2014 by trainees Ashley Powell, Sushan Ru, H.W., and P.S.; (**l**) examining and cataloging seedlings of 2018 families raised in pots in summer 2019 by trainees Duygu Demir (D.D.) and A.J.; (**m**) examining patchy red flesh in the selection “Red 6” in the breeding orchard in fall 2019; (**n**) tasting fruit in the breeding orchard in fall 2019 by trainees Fatih Topuz (F.T.), A.J., T.J., U.E., and D.D.; (**o**) harvesting fruit from selection “Bitter Shot” in fall 2020 by T.J. and A.J.; (**p**) pressing juice from several bitter-fruited selections and some bittersweet cultivar controls in fall 2019 by a group of trainees.

During the PWCabp’s operation from 2014 to 2021 through member participation, 13, 8, and 2 students in their undergraduate studies, graduate studies, or both, respectively, successfully gained experience and skills in many breeding program activities (Figure 1). Students gained hands-on experience in the goal-setting stage when they chose the crop for the program, identified the stakeholders the PWCabp aims to serve, and chose and defined target attributes (Figure 1). Students also gained experience in the breeding program stage of new genetic variation by successfully obtaining new germplasm to create a changing parent pool, planning and conducting crosses for more than 250 well-chosen parent combinations, collecting at least 1500 fruit from successful crosses, and stratifying more than 17,000 seeds until germination (Table 2, Figure 1). Hands-on experience in selection was achieved by students taking detailed phenotypic observations on which to base selection decisions on multiple occasions each year. Students also gained experience in obtaining and interpreting DNA information for seedling selection and for understanding the genetic composition of 18 new selections created by the program (Table S4). Further selection experience was gained by students identifying and propagating five promising selections generated by the breeding program.

## 3. Discussion

The PWCabp has facilitated hands-on learning for educating future plant breeders by providing the opportunity to investigate and implement practical solutions and make executive decisions for the program. Since establishment, the PWCabp has facilitated experiential learning for plant breeding students in a plethora of breeding program activities. CWR have been consistently used as a core physical resource, and DNA information has been sporadically used as a supplementary germplasm characterization and selection tool. The PWCabp has provided the opportunity for next-generation plant breeders to gain hands-on experience in key aspects of a breeding program in the safety of a learning environment. Competence in science-based knowledge and skills built on experimental results and experience is of the utmost importance for future plant breeders [6]. The PWCabp has facilitated experiential learning across a range of breeding operations, especially those involving hands-on activities with the plants themselves.

The goal-setting decision for cider apple to be the crop focus was informed by a combination of accessible diverse germplasm, an industry opportunity, and student preferences. Identification of the targeted market and stakeholders (backyard growers and hobbyists of the Palouse region) provided a goal with realistic focus and targets that could be readily expanded. Creating a paired cider apple logically connected with industry needs because U.S. cider makers already usually blend juice of specialty cider apples with that of culled fresh market fruit [33]. Because establishment of the PWCabp could occur only once, successive student cohorts could not participate in these initial steps. However, the PWCabp has facilitated ongoing experience in goal-setting via opportunities to reassess target attributes.

Chosen target attributes in the PWCabp are important for the target stakeholders, balancing novelty with functionality. Target attributes such as annual bearing and fire blight resistance focused on the primary flaws of specialty cider apple cultivars [24,25], while attributes such as red flesh color and high bitterness focused on a distinctive cider cultivar with unique characteristics, aligning with current industry trends [29]. Refinements to the 2014 list of target attributes such as adding the essential or enhancing designation in 2019 enabled more refined selection of parents and seedlings than in previous years. Traits such as yield, and leaves and bark appearance were not as well defined as other traits. Defining and quantifying specific trait thresholds would refine selection further while providing participants the opportunity to gain experience in the goal-setting stage of a breeding program.

**Figure 2:**
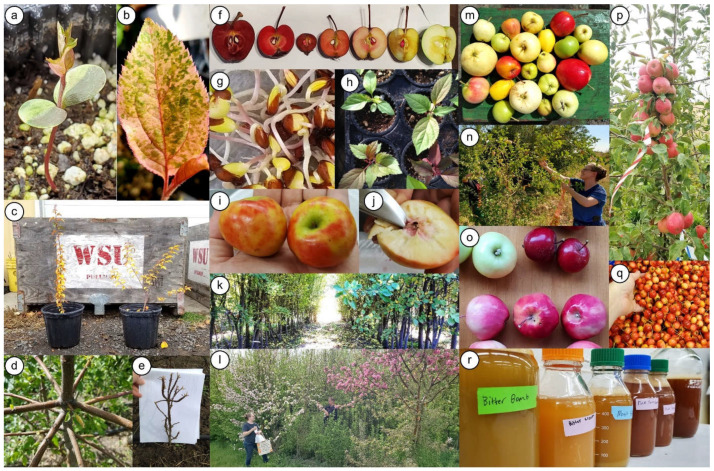
Some interesting phenotypes, parents, and selections arising in the Palouse Wild Cider apple breeding program. **Plant/tree phenotypes of interest**: (**a**) a variegated seedling given the moniker “Tiamat” that germinated in early 2020 from crossing a seedling of “Red Jade open-pollinated” with ‘Pendragon’; (**b**) “Tiamat” leaves have sectors of light and dark green, white/yellow, and pink/red; (**c**) two offspring of *M.* × *zumi* × ‘Wijcik McIntosh’ in fall 2017, showing segregation for the columnar branching habit of the paternal parent; (**d**) right-angled branching, commonly observed in offspring of ‘Kerr Crab’ and some *M. sieversii* parents; (**e**) a dwarfed seedling with many nodes and very short internodes, one of the many offspring from open pollination in 2013 of GMAL 2330 (*M. baccata*). **Red flesh and leaves**: (**f**) ‘Robert’s Crab’ (left), homozygous for the red flesh allele, has been the most common source of red flesh in the program, with each offspring inheriting one allele exhibiting one of many degrees of flesh redness, from the deep and vibrant red of “Red 11” and “Red 1” (second and third from left, respectively) to just redness at the core and seeds as in “Red 2” (second from right); (**g**) inheritance of the red-flesh allele is detectable in newly germinating seedlings by their pink radicles; (**h**) leaf color of program seedlings ranges from green for those with no red-flesh allele (top left) to some degree of redness for those with one red-flesh allele, having some to all leaves being reddish-green (top right and bottom left) to all leaves being vibrantly red (bottom right); (**i**) fruit in fall 2020 of the diploid selection “Red Io” resulting from the crossing in 2014 of an accession of *M. ioensis* and ‘Robert’s Crab’; “Red Io” has the typical red-green leaves of a heterozygous red-fleshed individual but unusually patchy skin blush and (**j**) redness inside the fruit only at the core and its few seeds. ***M. sieversii* germplasm base**: (**k**) hundreds of closely planted *M. sieversii* seedlings gave rise to numerous selections, both directly and in the next generation; (**l**) selections in bloom in spring 2021; “Bitter Cream” (left) was directly selected from the *M. sieversii* seedling block for phase 2, while “Pink Puma” (right) from the same block has been used as a parent, and both produce bittersweet fruit; (**m**) fruit phenotypic diversity of the *M. sieversii* seedling block observed in fall 2016. **Further selections**: (**n**) harvesting fruit of “Bitter Weeping” in fall 2020; (**o**) fruit of “Bitter Cream” (top left), “Red 11” (top right), and “Pink Puma” (bottom) in fall 2019; (**p**) “Big Blush” bearing its first crop of 91 fruit in summer 2016, exhibiting very short juvenility as it arose from open pollination of one of the 11 trees out of 800 in the *M. sieversii* seedling block that first began flowering and fruiting in 2013—a gamete-to-gamete cycle of just three years; (**q**) harvested tiny fruit of “Bitter Shot” from which juice was readily extracted in fall 2020; (**r**) juice collected in fall 2019 from pressed fruit of several bittersweet selections (as well as ‘Robert’s Crab’, far right).

Throughout the PWCabp’s operation, CWR featured prominently as parents or in parental genetic backgrounds. Through consistent use of diverse species of wild parents, students gained hands-on experience with introgression of CWR alleles. Increasing the number of seeds generated in recent years enabled more stringent selection than previous years and a higher probability that seedlings had desirable phenotypes. The genetic diversity found in CWR harbors many valuable alleles for important attributes such as short juvenility in apple [34], an attribute that was observed in some breeding program seedlings and exploited further by their subsequent use as parents. Enriching PWCabp germplasm for short juvenility enables students to select parents, make crosses, collect seeds, germinate and raise seedlings, and evaluate fruit of these new creations, all within the span of four years of undergraduate and/or graduate studies. Utilization of CWR in the PWCabp to date has yielded high phenotypic variation among program seedlings, and selection on such variation identified many seedlings with extreme (and often desirable) trait levels such as for branching architecture, bitterness, and flesh color (Figure 2).

The PWCabp has provided the opportunity for participants to gain experience in selection and the use of DNA information in a breeding program. Interpretation of SNP array data obtained for some parents and selections in the program successfully identified the paternal parent of most OP-derived selections and revealed accuracies and inaccuracies in intended parentage of seedlings. The incorrectly recorded maternal parent of “Big Blush” and its siblings was likely due to mislabeling in the field or an error in documenting which mother tree’s fruit was collected for the seeds of this family. “Red 5” and “Red 7” had phenotypically different fruit to each other but were revealed to be full siblings (‘Roberts Crab’ × ’Delicious’), visually highlighting for students the genetic reshuffling of parental genomes that occurs during sexual reproduction. Obtaining SNP array data on a seedling is expensive and therefore mostly cost-prohibitive for the program. In the future, the PWCabp would benefit from regular incorporation of efficient DNA profiling technology so that the genomic composition of a large number of program individuals can be better understood.

Using selection based on phenotypic and genotypic data, the PWCabp identified seedlings harboring multiple desirable attributes aligned with program targets. The use of marker-assisted seedling selection (MASS) helped identify superior seedlings, which contributes to breeding efficiency [20]. The much-reduced number of seedlings selected for field planting in 2019 and 2020 was likely due to high pathogen pressure; however, greenhouse-based selection was generally more stringent than in previous years. The program would benefit from increasing the number of seedlings planted in the greenhouse so that more seedlings pass phenotypic thresholds while selection remains stringent.

Beyond greenhouse seedlings, phenotypic selection and DNA-based information led to the identification of phase two selections, useful parents, and several other trees with interesting phenotypes among PWCabp germplasm (Figure 2). While DNA-based diagnostic tests useful to the PWCabp exist for many apple traits [20,35,36,37], the PWCabp would benefit from development of DNA tests for some traits specific to cider apples such as bitterness. In the future, SNP array information, such as that obtained for some parents, seedlings, and selections in the PWCabp, could be leveraged to determine functional genotypes at commercially important trait loci in PWCabp germplasm [38] or to discover and characterize trait loci for traits such as bitterness.

Sensory evaluations were key in identifying selections. The identification of high bitterness in several selections led to them being used as parents. However, the PWCabp would benefit from refining the sensory evaluation process. In the future, fruit maturity could be assessed using the iodine staining method and numerical scores from the 0–2 scale could be associated with instrumental measurements to improve robustness of phenotyping while retaining ease and rapidity in the field.

Although currently limited to a single site (the WSU Horticulture Center), establishment of a phase two selection trial will enable comparisons of tree and fruit performance against standard cider cultivars in an orchard setting. Future plans are to expand the trial to multiple locations to obtain data on tree performance in varied environments. This is an important step toward the commercialization stage, and while this stage has yet to come to fruition, the program has only been in operation for less than 10 years and breeding an apple cultivar from first crossing to commercial release can take 15–20 years [39,40]. With PWCabp phase two selections planted in 2021, an estimate of program selections being advanced to the commercialization stage and thus providing this missing experience is at least three years away.

The PWCabp has been a success in many aspects but faces some challenges and limitations. Promising cultivar candidates are beginning to be identified and second-generation germplasm has been used since 2017 to generate the third generation. Student engagement throughout the program has facilitated previously unavailable hands-on learning opportunities for more than 20 aspiring plant breeding professionals. Some challenges for the program are that it is not well known and funding is a limiting factor, with no funding directly tied to the program. More widespread awareness of the program would help attract students, and in turn, participation of a larger student body might enhance future funding opportunities. An area identified as lacking in the program is industry/stakeholder input. In the future, it is expected that the PWCabp’s students will regularly engage stakeholders through correspondence and questionnaires. Another challenge for the program is continuity and record keeping with new students continuously taking the helm. Transferring all previously gained knowledge to incoming new management can be a challenge faced by any breeding program, but it is exacerbated by the high turnover rate of personnel in the PWCabp. Therefore, maintaining detailed, comprehensible protocols and records would mitigate this challenge and ease the passing of the PWCabp management torch.

## 4. Materials and Methods

The PWCabp was officially established at WSU in Pullman, WA, in early 2014 for the WSU course HORT 495/503 “Fruit Breeding for the New Millennium”. Described below are the PWCabp’s approaches used to date, presented in the framework of the four generic breeding workflow stages described by Peace [19].

### 4.1. Goal-Setting

A suitable apple-derived crop, production region, and grower community were discussed by the first class of students to identify a set of targets that would not be too ambitious to enable a realistic focus. Once those were established, attributes to target were brainstormed and discussed, and a final list was agreed upon, with prompting guidance by the instructor and teaching assistant. These target attributes were periodically re-evaluated by subsequent student classes.

### 4.2. New Genetic Variation

Most of the parents used in 2013–2021 were trees growing in Block 17 of WSU’s Tukey Research Orchard (TRO) in Pullman, WA, that were planted in 2010 (the “Diversity Set” and the “Sieversii Site 6 and 9 Set”) described in the Introduction section. Each year, students chose parents for 200–500 crossings, which were compiled into a spreadsheet. In some years, 2000–4000 crossings were randomly chosen from the spreadsheet; however, in many years, opportunity was limited by the trees that were flowering (some trees were biennial and some affected by disease) and labor availability, and therefore crosses were chosen to efficiently use available resources. The breeding program’s first year of families, derived from 2013 pollinations, were entirely the result of open pollinations because the program’s objectives had not yet been defined. Thereafter, controlled crossing was performed by students to create new genetic variation and gain experience in planning crossing, collecting pollen, and performing controlled pollination. Exceptions to controlled crossing were sets of seeds obtained from open pollination-derived fruit of intriguing cider apple cultivars and program seedlings to help boost seed numbers where desired.

Each year in late fall, students collected fruit from the previous spring’s crossings and extracted the seeds. Over winter/early spring, students stratified seeds in preparation for germination. Each family of seeds was placed in a labeled Petri dish with a moistened absorbent liner. Seeds were then stored in a refrigerator at 4 °C and checked once a week for adequate moisture and fungal infection. Once germinated, seedlings were planted in 4 × 8 cone trays in a greenhouse with a 16 h photoperiod and kept at 18–28 °C.

### 4.3. Selection

Selection was conducted in the greenhouse and field each season. Once each year’s seeds obtained from crosses had germinated, those displaying the most vigorous growth were selected for greenhouse planting. In families intended to create red-fleshed apples, only seedlings showing the red phenotype were selected for planting. During the spring, seedlings were raised in the greenhouse. Selection in the greenhouse involved culling first the dead and poor-vigor seedlings then removing those exhibiting disease symptoms (especially for powdery mildew and apple cedar rust) with detailed observations recorded on each family in 2019 and 2020. At each selection step, numbers of seedlings for each family were recorded.

Genotyping of program seedlings was conducted in 2014 primarily for students to gain marker-assisted seedling selection (MASS) experience. Genotyping was conducted for most of the 800 greenhouse-planted seedlings of the 2013 families derived from open pollination, with a target number for field-planting of 400. A subset of 152 seedlings (2–16 seedlings per family depending on family size) were initially genotyped with two DNA tests to determine typical segregation. Remaining seedlings of families in which desirable genotypes were segregating were then genotyped with one or both DNA tests. The DNA tests were Ma-indel [18] and Rf-SSR (forward primer: /5HEX/GTATGGCCTCCAATGTTTCC, reverse primer: GGTCAAATGGGATTTTAGGC). Leaf samples were collected, and DNA was extracted via the silica bead method [41]. Amplification was performed on a Tetrad^®^ 2 thermal cycler (BIO-RAD Laboratories Inc., Hercules, CA, USA) using the protocol as in Ru et al. [18]. Amplicons were separated with an Applied Biosystems^®^ 3730 DNA Analyzer and scored using GeneMarker^®^ software. For Ma-indel, the desirable seedling genotype was heterozygous Ma|ma, which was considered to be associated with sufficient fruit acidity and high phenolic levels, the latter based on the deduction that the Ma allele was associated with low phenolic levels, ma with high phenolic levels, and the high-phenolics allele being dominant [42,43]. For Rf-SSR, the desirable genotypes were Rf|Rf and Rf|rf, associated with at least some skin blush, considered at that time to provide some benefit to the cider product, although it was later decided that skin color does not significantly contribute to targeted cider apple attributes. For both DNA tests, alleles of unknown effect, especially new alleles from wild parents, were considered desirable. Seedlings possessing desirable alleles were selected for field planting. As this season’s seedlings were genotyped early in the year (April), disease symptoms were not yet showing, such that the only seedlings not considered for genotyping and subsequent field planting were those that had not germinated or had poor vigor. In subsequent years, genotyping was not conducted because numbers of seedlings after phenotypic culling in the greenhouse were within field maintenance capacity and available DNA tests were not considered appropriate for further culling.

Seedlings selected for field planting were planted in the phase one field trial in block 17 of TRO from 2014 to 2015; however, 2016–2018 seedlings selected for field planting were transplanted and kept in two-gallon plastic pots due to an imminent relocation of the program’s field operations. Field-based phenotypic observations on traits such as fruit size, flesh color, phenolic compound amounts, juvenility duration, fire blight resistance, bearing habit, and branch angle were recorded on field-planted seedlings over successive years, and further selection was applied on the basis of tree performance, branch architecture, and fruit quality. Because fruit quality is a main driver in breeding cider apples, this aspect was assessed by conducting sensory evaluations after seedlings flowered and fruit became available. From August to October each year, fruit maturity was checked weekly using simple sensory indices such as fruit drop, color, seed maturity, and sweetness. Once fruit was mature, enough fruit were collected to conduct an evaluation and a minimum of three participants came to a consensus score for traits on a 0–2 scale (increments of 0.5). Fruit traits evaluated on this scale were sweetness, acidity, bitterness, astringency, firmness, and crispness. Other fruit traits recorded were size, shape, and flesh color. In 2019, the primary location of PWCabp operations involving physical plants was moved from TRO to the WSU Horticulture Center, and students redesigned and implemented the phase one trial for the new site. Irrigation for the system was also designed by students. In 2020 and 2021, scions of selected seedlings were collected and grafted onto G.11 rootstock using the whip and tongue technique. Students established a phase two trial row at the WSU Horticulture Center and grafted trees were planted in spring of 2021. Trees were planted at three-foot spacing on a five-wire vertical trellis system with drip irrigation.

Some selections from the program and cultivars used as parents had leaf samples collected, had DNA extracted [44], and were run on the Illumina 20K apple SNP array [45]. DNA profile data obtained for each sampled tree were used to confirm identity in the case of parents, to confirm or determine parentage in the case of seedlings, and to identify affinities to DNA profiles of various *Malus* species accessions via simple genotype-matching in Microsoft Excel.

### 4.4. Commercialization

No activities were conducted involving advancement of selections to the stage of commercialization because of the young age of the breeding program.

## 5. Conclusions

This report was a description of the first nine years of an unusual breeding program focused on student training and use of CWR and DNA information. The PWCabp’s approaches, outcomes, and facilitation of hands-on learning for plant breeding students were described while highlighting ongoing use of diverse germplasm and some incorporation of DNA information. For the future, the program aims to secure greater funding and advance selections to the commercialization stage. Because of the highly diverse *Malus* germplasm base used in the program, unique and desirable new germplasm is the expected breeding outcome. The PWCabp will continue to provide students with education and experience that they can take to other breeding programs, enabling them to contribute to addressing global issues such as climate change and food insecurity through plant breeding.

## Figures and Tables

**Table 1:** List of target traits in the student run PWCabp, including trait levels and their designation as essential or enhancing. Essential attributes are those that represent the minimum level of a trait for a cultivar produced in the program, while enhancing attributes are those that are above and beyond targeted trait levels and can boost cultivar value. Some traits possessed a second, more enhancing level (2×) beyond the basic enhancing level of these traits (1×).

Trait	2014 Goal	2019 Goal	Trait Level	Essential or Enhancing
Flesh color	X	X	Heterozygous for red flesh	Enhancing 1×
	X	X	Homozygous for red flesh	Enhancing 2×
Fruit size		X	>40 mm (i.e., at least golf ball size)	Essential
		X	>60 mm (i.e., at least tennis ball size)	Enhancing
Phenolic compound amount	X	X	1.0–1.5 on a 0–2 sensory evaluation scale (bitter after blend)	Essential
	X	X	>1.5 on a 0–2 sensory evaluation scale (extremely bitter but still blends well)	Enhancing
Juiciness		X	200 mL juice per lb fresh fruit	Essential
		X	>275 mL juice per lb fresh fruit	Enhancing
Seediness	X		Not seedy	Enhancing
Nutritional components	X		Nutritional boost	Enhancing
Juvenility duration	X	X	Fruit in fifth leaf	Essential
	X	X	Fruit in fourth leaf	Enhancing 1×
	X	X	Fruit in third leaf	Enhancing 2×
Fire blight resistance	X	X	Not susceptible	Essential
	X	X	Highly tolerant	Enhancing 1×
	X	X	Fully resistant	Enhancing 2×
Powdery mildew resistance	X	X	Not susceptible	Essential
Cedar apple rust resistance		X	Not susceptible	Essential
		X	Highly tolerant	Enhancing 1×
		X	Fully resistant	Enhancing 2×
Fruit-bearing habit	X	X	Not tip bearing	Essential
		X	Clearly spur-bearing type	Enhancing
Annual bearing tendency	X	X	Not biennial bearing	Essential
Leaves and bark	X	X	Novel shape, color, texture, etc.	Enhancing
Branch angle	X	X	>70°	Enhancing 1×
	X	X	90°	Enhancing 2×
Yield	X	X	High yielding	Enhancing

**Table 2:** Number of flowers pollinated including wild-background parents used, largest family created, and highlighted outcomes from crosses for 2013–2021. OP = open pollinated: no flower emasculation or intentional application of particular parental pollen. n.d. = no data recorded.

Year of Crossing	No. Flowers Pollinated	No. *M. sieversii* Parents	No. Other Wild Parents Used	No. Hybrid Wild × Non-Wild Parents Used	No. Non-Wild Parents Used	No. of Families Attempted/ Successful	No. Attempted Families with Wild Background	Largest Family Created (no. Seeds)	No. Seeds Created *	No. Seeds Greenhouse Planted and Raised	No. Seedlings Planted in Field	Flowering and Fruiting Outcomes of Crosses
2013	OP	7	5	4	5	30/30	24	‘Kerr Crab’ OP (337)	1700	800	350	Flowering and fruiting since 2016: “Big Blush”, “Yellow Tiny”, “Red-Yellow Tiny”, “Orange Tiny”, “Stripey”
2014	574	2	6	1	19	69/18	66	‘Kerr Crab’ × PRI E14-32 (157)	1028	865	530	Flowering and fruiting since 2018: “Red 1” and “Red 2”; flowering and fruiting from 2019: “Red 3” to “Red 16” (except “Red 14”)
2015	2867	0	6	3	14	79/21	75	‘Robert’s Crab’ OP (311)	512	n.d.	n.d.	One seedling flowering since 2021
2016	4146	0	8	0	2	25/15	25	‘Golden Delicious’ × *M.* × *zumi* (450)	1612	n.d.	n.d.	Two seedlings with *M.* × *zumi* mother flowering since 2021
2017	5879	4	5	2	3	29/20	29	Golden ‘Delicious’ × “Big Blush” (356)	1360	858	425	**
2018	4319	0	6	2	1	24/19	24	“Yellow Tiny” × “Big Blush” (615)	3281	976	243	**
2019	>2500	1	0	11	19	23/20	20	“Red 1” × ‘Wickson Crab’ (415)	3727	1297	148	**
2020	1916	1	0	13	6	27/18	22	“Most Bitter” × “Red 11” (153)	3054	757	130	**
2021	1774	4	1	3	4	11/7	11	‘King-ston Black’ × “Pink Puma” (115)	1606	***	***	***

* Number includes open pollination-derived seeds. ** Seedlings from this year have not yet fruited. *** Seeds are in stratification; no data for greenhouse or field planting.

## Data Availability

The data presented in this study are available within the article and its Supplementary Materials.

## Supplementary Materials

The following are available online at https://www.mdpi.com/article/10.3390/plants11040517/s1, Figure S1: Phase one field trial at the WSU Horticulture Center designed by students for the PWCabp (Figure S1**a**). Moving from Tukey Research Orchard (TRO) to the WSU Horticulture center provided an opportunity to redesign the phase one field trial. The new design includes the switch to growing trees in pots instead of in the ground. Growing trees in pots has several advantages over planting in the ground. One advantage is plant mobility. Not being rooted in the ground allows easy rearrangement (working with limited space makes this valuable) and culling of trees that do not meet target thresholds. Drip irrigation (Figure S1**b**) provides more efficient application of water while being relatively inexpensive compared to overhead irrigation systems. There are drawbacks to growing trees in pots instead of in the ground. Fabric pots are not permanent and can be expensive to buy and replace. Soil is required to fill the pots, adding to the expense, and less natural precipitation is used by potted trees. Overall, the redesigned phase one field trial is a more manageable system than the previous in-ground site at TRO. Future plans are to expand the field trial area to allow increased seedling numbers. Table S1: List of species genetics represented by PWCabp parents of seeds created from each year’s crossings. In each year, most species were represented in multiple parental combinations. Table S2: Greenhouse culling of seedlings of PWCabp 2019 families to result in vigorous, disease symptom-free seedlings for field planting. OP = Open pollinated: no flower emasculation or intentional application of particular parental pollen. Note: None of the families with “Red 2” as a parent gave red seedlings as expected so there was no culling for non-reds in these families; however, because an independent check in 2020 indicated that “Red 2” OP seeds indeed segregate 1:1 for the red phenotype (results not shown), we suspect that the “Red 2” pollinations in 2019 were unsuccessful. Table S3: Greenhouse culling of seedlings of PWCabp 2020 families to result in vigorous, disease symptom-free seedlings for field planting. OP = Open pollinated: no flower emasculation or intentional application of particular parental pollen. Table S4: Genetic features of seedlings unique to the PWCabp selected for advanced evaluation and/or used as parents. Determinations and estimations were based on analyses of 20K SNP array data. “Bittersweet” indicates high bitterness and low acidity. “Very short juvenility” indicates flowering in 3rd leaf while “short juvenility” indicates 4th leaf. “Small fruit” indicates at least golf ball size. OP = Open pollinated: no flower emasculation and intentional application of particular parental pollen.
