# Supplementary material for: As It Stands: The Palouse Wild Cider Apple Breeding Program
Source: Plants (Basel). 2022 Feb 14;11(4):517. doi: 10.3390/plants11040517 (PMC8877849; doi:10.3390/plants11040517)
Supplement: Supplementary file 1 [file plants-11-00517-s001.zip › Figure S1 Phase 1 design submitted.pdf]

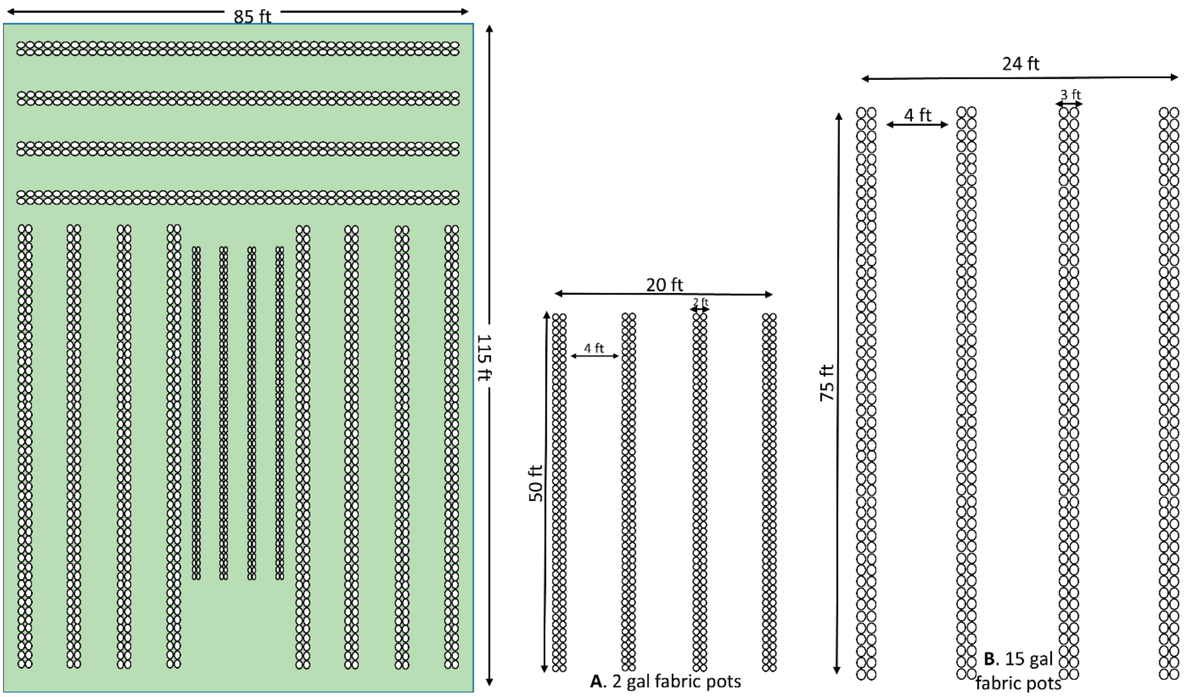
85 ft
115 ft
20 ft
4 ft
2 ft
50 ft
A. 2 gal fabric pots
24 ft
4 ft
3 ft
75 ft
B. 15 gal fabric pots

Figure S1a.

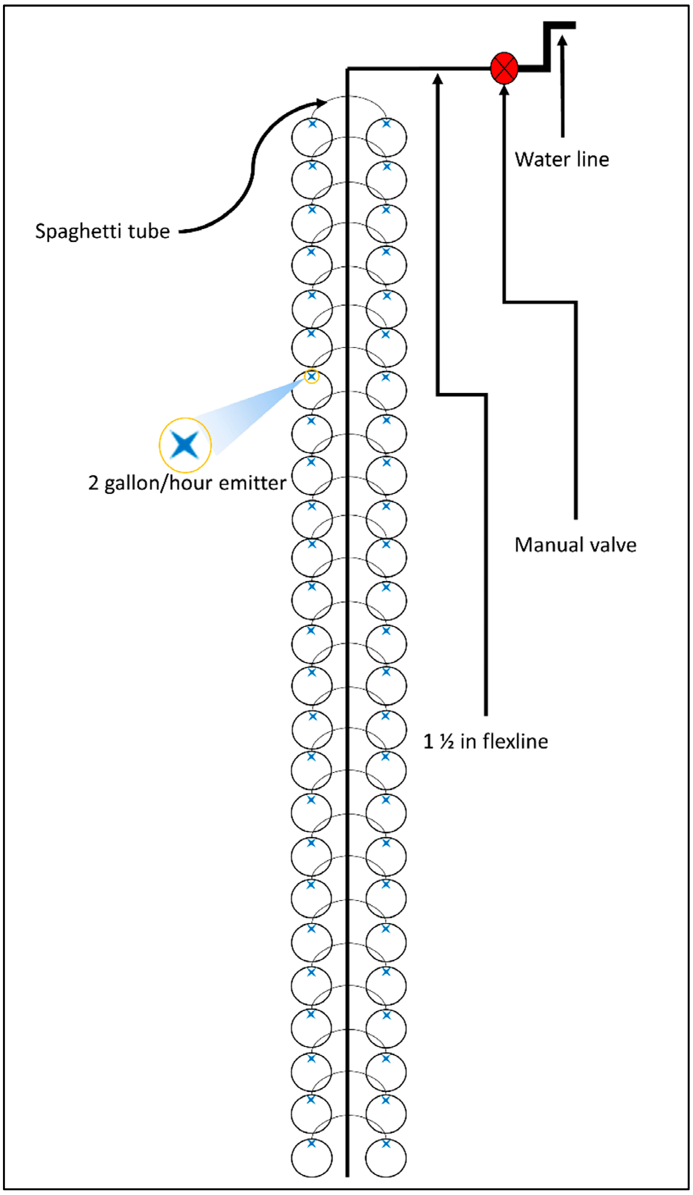

Figure S1b.

Figure S1. Phase one field trial at the WSU Horticulture Center designed by students for the PWCabp (Figure S1a). Moving from Tukey Research Orchard (TRO) to the WSU Horticulture center provided an opportunity to redesign the phase one field trial. The new design includes the switch to growing trees in pots instead of in the ground. Growing trees in pots has several advantages over planting in the ground. One advantage is plant mobility. Not being rooted in the ground allows easy rearrangement (working with limited space makes this valuable) and culling of trees that do not meet target thresholds. Drip irrigation (Figure S1b) provides more efficient application of water while being relatively inexpensive compared to overhead irrigation systems. There are drawbacks to growing trees in pots instead of in the ground. Fabric pots are not permanent and can be expensive to buy and replace. Soil is required to fill the pots, adding to the expense, and less natural precipitation is used by potted trees. Overall, the redesigned phase one field trial is a more manageable system than the previous in-ground site at TRO. Future plans are to expand the field trial area to allow increased seedling numbers.
