# Supplementary material for: As It Stands: The Palouse Wild Cider Apple Breeding Program
Source: Plants (Basel). 2022 Feb 14;11(4):517. doi: 10.3390/plants11040517 (PMC8877849; doi:10.3390/plants11040517)
Supplement: Supplementary file 1 [file plants-11-00517-s001.zip › Table S1 species represented by parents - submitted.pdf]

Table S1. List of species genetics represented by PWCabp parents of seeds created from each year’s crossings. In each year, most species were represented in multiple parental combinations.

	**Species’ genetics represented in PWCabp parents**								
**Year**	**2013**	**2014**	**2015**	**2016**	**2017**	**2018**	**2019**	**2020**	**2021**
**Species**	*M. baccata*<br>*M. domestica*<br>*M. floribunda*<br>*M. prunifolia*<br>*M. sieversii*<br>*M. ×soulardii*	*M. baccata*<br>*M. domestica*<br>*M. floribunda*<br>*M. ioensis*<br>*M. ×micromalus*<br>*M. prunifolia*<br>*M. sieversii*<br>*M. ×atrosanguinea*<br>*Malus* spp.	*M. domestica*<br>*M. ioensis*<br>*M. ×micromalus*<br>*M. prunifolia*<br>*M. sieboldii*<br>*M. sieversii*	*M. baccata*<br>*M. domestica*<br>*M. floribund,*<br>*M. fusca*<br>*M. ×robusta*<br>*M. ×zumi*	*M. baccata*<br>*M. domestica*<br>*M. ioensis*<br>*M. sieboldii*<br>*M. sieversii*<br>*M. ×zumi*	*M. baccata*<br>*M. domestica*<br>*M. ioensis*<br>*M. prunifolia*<br>*M. sieversii*<br>*M. ×zumi*<br>*Malus* spp.	*M. baccata*<br>*M. domestica*<br>*M. ioensis*<br>*M. prunifolia*<br>*M. sieversii*	*M. baccata*<br>*M. domestica*<br>*M. ioensis*<br>*M. prunifolia*<br>*M. sieversii*	*M. domestica*<br>*M. ioensis*<br>*M. prunifolia*<br>*M. sieversii*
