# Supplementary material for: As It Stands: The Palouse Wild Cider Apple Breeding Program
Source: Plants (Basel). 2022 Feb 14;11(4):517. doi: 10.3390/plants11040517 (PMC8877849; doi:10.3390/plants11040517)
Supplement: Supplementary file 1 [file plants-11-00517-s001.zip › Table S2 2019 Seedling culling - submitted.pdf]

Table S2. Greenhouse culling of seedlings of PWCabp 2019 families to result in vigorous, disease symptom-free seedlings for field planting. OP = Open pollinated: no flower emasculation or intentional application of particular parental pollen. Note: None of the families with "Red 2" as a parent gave red seedlings as expected so there was no culling for non-reds in these families; however, because an independent check in 2020 indicated that "Red 2" OP seeds indeed segregate 1:1 for the red phenotype (results not shown), we suspect that the "Red 2" pollinations in 2019 were unsuccessful.

**Parents of family**		**Estimated proportion wild background in seedlings of family (sieversii, other species, domestica)**	**Total**	**No. seedlings culled and reason**				**Kept, field-planted**	**Proportion culled**
**Mother**	**Father**			**Powdery mildew**	**Apple cedar rust**	**Poor vigor**	**Dead**		
Breakwell's Seedling	N3-4b-25 ("Big Blush")	**25%**, 75%	170	74	83	1	3	9	95%
Liberty	N3-4b-25 ("Big Blush")	**25%**, 75%	126	49	51	17	3	6	95%
Cox's Orange Pippin	N3-4b-25 ("Big Blush")		78	35	35	0	0	8	90%
Red Summer Rambo	N3-4b-25 ("Big Blush")	**25%**, 75%	18	5	3	6	0	4	78%
Hewe's Crab	N3-4b-25 ("Big Blush")		12	4	3	3	1	1	92%
Delicious	"Big Blush" and "Red 2"		157	80	68	3	1	5	97%
Delicious	N3-4b-25 ("Big Blush")	**25%**, 75%	22	9	8	0	0	5	77%
Delicious	S4-3a-9 ("Red 2")	**28%**, 7%, 65%	9	4	5	0	0	0	100%
N3-4b-25 ("Big Blush")	S4-3a-9 ("Red 2")	**53%**, 7%, 40%	91	6	39	35	1	10	89%
S4-4a-22 ("Red Io")	OP		2	0	0	0	1	1	50%
S4-3a-26 (Red Jade OP)	Bramtot		28	0	23	0	2	3	89%
S4-3a-26 (Red Jade OP)	Calville Rouge		9	0	4	2	1	2	78%
S4-3a-26 (Red Jade OP)	Frequin		8	3	0	1	1	3	63%
S3-4b-25 (E14-32 OP)	S4-3a-9 ("Red 2")	**28%**, 7%, 65%	21	4	5	0	3	9	57%

S3-4b-25 (E14-32 OP)	N3-4b-25 ("Big Blush")	**25%**, 75%	16	4	11	0	1	0	100%
S3-4b-25 (E14-32 OP)	S4-3a-25 ("Red 1")	**12%**, 25%, 63%	1	1	0	0	0	0	100% *
S3-4b-10 ("Bitter Shot")	Bramtot	23%, 77%	82	26	25	8	7	16	81%
S3-4b-10 ("Bitter Shot")	Amere de Berthecourt	23%, 77%	77	21	44	1	5	6	92%
S3-4b-10 ("Bitter Shot")	Frequin	23%, 77%	65	4	23	5	24	9	86%
S3-4b-10 ("Bitter Shot")	Calville Rouge	23%, 77%	22	7	7	0	3	5	77%
S4-3a-25 ("Red 1")	Wickson Crab		132	79	4	31	5	13	90% *
S4-3a-25 ("Red 1")	Gros Launet	**13%**, 25%, 62%	100	37	23	11	3	26	74% *
Delicious	"Red 1"	**13%**, 25%, 62%	25	16	5	1	0	3	88% *
PRI 1773-6	S1-1b-19 (red-fleshed site 6 *sieversii*)	**50%**, 50%	10	4	3	0	0	3	70% *
PRI 1773-6	Pendragon		8	4	0	1	0	3	62% *
S4-3a-26 (Red Jade OP)	Pendragon		6	0	0	0	3	3	50% *
S4-3a-6 ("Red 6")	OP		2	2	0	0	0	0	100% *
Total			1297	478	472	126	68	153	Ave. tot. % cull
Proportion				36%	36%	10%	5%	12%	88%

*Only red seedlings selected for this family. Selected as newly germinated seeds with a red radicle (Figure 2g). Expected ratio of red to non-red phenotypes in a family generated with one parent heterozygous for the red flesh allele is 1:1.
