# Supplementary material for: As It Stands: The Palouse Wild Cider Apple Breeding Program
Source: Plants (Basel). 2022 Feb 14;11(4):517. doi: 10.3390/plants11040517 (PMC8877849; doi:10.3390/plants11040517)
Supplement: Supplementary file 1 [file plants-11-00517-s001.zip › Table S3 2020 Seedling culling - submitted.pdf]

Table S3. Greenhouse culling of seedlings of PWCabp 2020 families to result in vigorous, disease symptom-free seedlings for field planting. OP = Open pollinated: no flower emasculation or intentional application of particular parental pollen.

Parents of family		Estimated proportion wild background in seedlings of family (**sieversii**, other species, domestica)	Total No. seedlings in family	No. seedlings culled and reason				Kept, field-planted	Proportion culled
Mother	Father			Powdery mildew	Apple cedar rust	Poor vigor	Dead		
WSU cv2	GMAL 2251	**50%**, 50%	52	39	0	0	0	13	75%
WSU cv2	GMAL 4002.h A-4/5 0.2 ("Bitter Cream")	**50%**, 50%	28	16	3	0	0	9	68%
WSU cv2	N3-4a-26 ("Bitter Bomb")	**50%**, 50%	12	4	4	0	0	4	66%
WSU cv2	S3-1a-24 ("Bitter Weeping")	**50%**, 50%	9	4	0	0	0	5	44%
WSU cv1	GMAL 2251	**50%**, 50%	71	43	3	10	1	14	80%
WSU cv1	GMAL 4002.h A-4/5 0.2 ("Bitter Cream")	**50%**, 50%	66	45	9	0	0	12	82%
WSU cv1	N3-4a-26 ("Bitter Bomb")	**50%**, 50%	69	37	21	1	4	6	91%
WSU cv1	S3-1a-24 ("Bitter Weeping")	**50%**, 50%	32	24	0	1	1	6	81%
S4-4a-22 ("Red Io")	GMAL 2251	**62%**, 25%, 13%	2	0	0	0	0	2	0%
GMAL 2251	S4-1b-20 ("Red 11")	**63%**, 9%, 28%	27	15	5	0	1	6	78% *
GMAL 2251	S4-2b-7 ("Red 8")	**63%**, 25%, 12%	19	5	5	0	0	9	53% *
S3-4b-10 ("Bitter Shot")	OP		16	5	2	0	1	8	50% *
S4-4a-22 ("Red Io")	OP		7	4	1	0	1	1	86% *
S4-3b-7 ("Red 5")	OP		5	3	0	0	0	2	60% *
S3-4b-10 ("Bitter Shot")	OP		342	219	59	37	5	22	93%
Total			757	463	112	49	14	119	Ave. tot. % cull
Proportion				61%	15%	6%	2%	16%	84%

*Only red seedlings selected for this family. Selected as newly germinated seeds with a red radicle (Figure 2g). Expected ratio of red to non-red phenotypes in a family generated with one parent heterozygous for the red flesh allele is 1:1.
