# Supplementary material for: As It Stands: The Palouse Wild Cider Apple Breeding Program
Source: Plants (Basel). 2022 Feb 14;11(4):517. doi: 10.3390/plants11040517 (PMC8877849; doi:10.3390/plants11040517)
Supplement: Supplementary file 1 [file plants-11-00517-s001.zip › Table S4 Selections, CWR use - submitted.pdf]

Table S4. Genetic features of seedlings unique to the PWCabp selected for advanced evaluation and/or used as parents. Determinations and estimations were based on analyses of 20K SNP array data. "Bittersweet" indicates high bitterness and low acidity. "Very short juvenility" indicates flowering in 3rd leaf while "short juvenility" indicates 4th leaf. "Small fruit" indicates at least golf ball size. OP = Open pollinated: no flower emasculation and intentional application of particular parental pollen.

Selection	Moniker	Distinctive attributes (<u>enhancing</u>, **essential**, *undesirable*, interesting/different)	Origin	Parentage, intended	Parentage, determined		*Malus* species composition, estimated*
					Mother	Father	
GMAL 4002.h A-4/5 0.2 (PWC N1-4b-14)	Bitter Cream	**bittersweet and non-astringent**, **small fruit**, creamy-yellow blushless skin, *biennial bearing*	Kazakhstan "site 6"	GMAL 3785.b (*M. sieversii*) × pollen pool D-4/5 (*M. sieversii*)	GMAL 4002.h (KAZ 95 18-02P-33, PI 657083)	unknown sieversii of site 6's pollen pool A-4/5	100% *sieversii*
GMAL 3762.h A-06 0.4 (PWC N1-4b-2)	Pink Puma	<u>red flesh and tree</u>, **bittersweet**, **non-astringent**, **small fruit**, *biennial bearing*, *PM susceptible*	Kazakhstan "site 9"	GMAL 3762.h (*M. sieversii*) × pollen pool C-06 (*M. sieversii*)	GMAL 3762.h (KAZ 95 05-01P-3.3, PI 657058)	GMAL 3781.c (KAZ 95 05-01P-22.2, PI 657064)	100% *sieversii*
PWC N3-4b-25	Big Blush	<u>very short juvenility</u>, <u>right-angled branches</u>, **small fruit** (larger and blushed compared to sieversii parent), *only slightly bitter*, *slightly astringent and acidic*	PWCabp 2013 OP families	GMAL 3614.a A-4/5 0.2 (*M. sieversii*, Kazakhstan "site 9") OP	unknown sieversii (not GMAL 3614.a A-4/5 0.2)	Haralson	50% *domestica*, 50% *sieversii*
PWC N3-4b-12	Yellow Tiny	<u>very short juvenility</u>, yellow-skinned without blush, *tiny fruit*, *astringent*, *not bitter*	PWCabp 2013 OP families	GMAL 1890 (*M. prunifolia*) OP	GMAL 1890	unknown tiny-fruited in baccata group	50% *prunifolia*, 50% *baccata*
PWC N3-3b-7	Red-Yellow Tiny	<u>very short juvenility</u>, yellow-skinned with blush, *tiny fruit*, *astringent*, *not bitter*	PWCabp 2013 OP families	GMAL 1890 (*M. prunifolia*) OP	GMAL 1890	unknown tiny-fruited in baccata group	50% *prunifolia*, 50% *baccata*
PWC N3-2b-16	Orange Tiny	<u>very short juvenility</u>, orange-skinned without blush, *tiny fruit*, *astringent*, *not bitter*	PWCabp 2013 OP families	GMAL 2330 (*M. baccata*) OP	GMAL 2330	unknown tiny-fruited in baccata group	100% *baccata*
PWC S3-3b-1	Stripey	<u>very short juvenility</u>, yellow-skinned with prominent stripes, very crisp, *tiny fruit*, *biennial bearing*, *not bitter*	PWCabp 2013 OP families	Kerr OP	Kerr	White Angel	56% *domestica*, 44% *baccata* group
PWC S3-1a-24	Bitter Weeping	**bittersweet and non-astringent**, **small fruit**, *tip bearing*	PWCabp 2013 OP families	GMAL 2251 OP	GMAL 2251	unknown sieversii	100% *sieversii*
PWC N3-4a-26	Bitter Bomb	**bittersweet and non-astringent**, **small fruit**, *biennial bearing*	PWCabp 2013 OP families	GMAL 3614.a A-4/5 0.2 (*M. sieversii*) OP	unknown sieversii (not GMAL 3614.a A-4/5 0.2)	unknown sieversii	100% *sieversii*
PWC S3-4b-10	Bitter Shot	**bittersweet and non-astringent**, *tiny fruit*, *biennial bearing*	PWCabp 2013 OP families	PRI E14-32 OP	PRI E14-32	unknown tiny-fruited, very close to White Angel	55% *domestica*, 44% *baccata* group, 1% *floribunda*

Table S4 cont'd.

Selection	Moniker	Distinctive attributes (enhancing, **essential,** *undesirable,* interesting/different)	Origin	Parentage, intended	Parentage, determined		*Malus* species composition, estimated*
					Mother	Father	
PWC S4-3a-25	Red 1	short juvenility, red flesh and tree, heavy annual bearing, *tiny fruit*, *astringent*, *not bitter*, *fire blight susceptible*	PWCabp 2014 OP families	Robert's Crab OP	Robert's Crab	unknown tiny-fruited in baccata group	50% *baccata*, 25% *sieversii*, 25% *domestica*
PWC S4-3a-9	Red 2	short juvenility, red flesh at core only, red tree, right-angled branches, **small fruit**, dessert-like taste, *not bitter*	PWCabp 2014 families	Novosibirski Sweet x Robert's Crab	Robert's Crab	Novosibirski Sweet	56% *sieversii*, 31% *domestica*, 13% *prunifolia*
PWC S4-3b-7	Red 5	medium-sized fruit, red flesh and tree, dessert-like taste, *biennial bearing*, *not bitter*	PWCabp 2014 OP families	Robert's Crab OP	Robert's Crab	Delicious	75% *domestica*, 25% *sieversii*
PWC S4-3a-6	Red 6	red flesh in patches, red tree, **small fruit**, dessert-like, *not bitter*, *fire blight susceptible*	PWCabp 2014 families	Novosibirski Sweet x Robert's Crab	Robert's Crab	Novosibirski Sweet	56% *sieversii*, 31% *domestica*, 13% *prunifolia*
PWC S4-4b-25	Red 7	red flesh and tree, dessert-like, *tiny fruit*, *not bitter*	PWCabp 2014 OP families	Robert's Crab OP	Robert's Crab	Delicious	75% *domestica*, 25% *sieversii*
PWC S4-2b-7	Red 8	red flesh and tree, **small fruit**, *biennial bearing*, *astringent*, *not bitter*	PWCabp 2014 OP families	Robert's Crab OP	Robert's Crab	unknown tiny-fruited in baccata group	50% *baccata*, 25% *sieversii*, 25% *domestica*
PWC S4-1b-20	Red 11	deep red flesh, red tree, right-angled branches, **small fruit**, dessert-like taste, very crisp, *not bitter*, *fire blight susceptible*	PWCabp 2014 families	Robert's Crab x Kerr	Robert's Crab	Kerr	56% *domestica*, 25% *sieversii*, 19% *baccata*
PWC S4-4a-22	Red Io	red flesh at core only, red tree, **small fruit**, slightly lobed leaves, *astringent*, *somewhat spiny spurs*, *not bitter*	PWCabp 2014 families	GMAL 2939 (*M. ioensis*) × Robert's Crab	GMAL 2939	Robert's Crab	50% *ioensis*, 25% *sieversii*, 25% *domestica*

* *Malus* species composition based on estimations that: PRI E14-32 has one *domestica* parent (unknown identity) and other parent ('Co-op 9') is 63/64 *domestica*, 1/64 *floribunda*; 'Dolgo' (parent of 'Kerr') and 'White Angel' are ~3/4 *baccata*, ~1/4 *domestica*; 'Robert's Crab' is ~1/2 *sieversii*, ~1/2 *domestica*; 'Novosibirski Sweet' is ~5/8 *sieversii*, ~1/4 *prunifolia*, ~1/8 *domestica*.
